# Supplementary material for: PRMT5 and CDK4/6 inhibition result in distinctive patterns of alternative splicing in melanoma
Source: PLoS One. 2023 Nov 2;18(11):e0292278. doi: 10.1371/journal.pone.0292278 (PMC10621831; doi:10.1371/journal.pone.0292278)
Supplement: S1 Table — (DOCX) [file pone.0292278.s001.docx]

S1 Table. Numbers of reads, library size and HTSeq gene count for samples from CHL1 and A375 cells.

| **Sample** | **Number of Read Pairs (Picard)** | **Estimated Library Size (Picard)** | **Expressed gene** |
| --- | --- | --- | --- |
| A375_Control_72hours_replicate1 | 29445687 | 130995840 | 23456 |
| A375_Control_72hours_replicate2 | 31607087 | 125259365 | 22953 |
| A375_CDK4/6i_72hours_replicate1 | 31321381 | 98816131 | 23660 |
| A375_CDK4/6i_72hours_replicate2 | 32851773 | 115445154 | 23726 |
| A375_PRMT5i_72hours_replicate1 | 28600785 | 93590565 | 23511 |
| A375_PRMT5i_72hours_replicate2 | 29683128 | 87086633 | 22675 |
| A375_Control_6days_replicate1 | 54976837 | 339099090 | 24596 |
| A375_Control_6days_replicate2 | 47990586 | 337523343 | 24723 |
| A375_CDK4/6i_6days_replicate1 | 49311674 | 179617096 | 25278 |
| A375_CDK4/6i_6days_replicate2 | 48510771 | 111721929 | 25750 |
| CHL1_Control_72hours_replicate1 | 46071287 | 283848084 | 23331 |
| CHL1_Control_72hours_replicate2 | 44361715 | 297983024 | 23365 |
| CHL1_CDK4/6i_72hours_replicate1 | 44430155 | 219996906 | 23586 |
| CHL1_CDK4/6i_72hours_replicate2 | 44359734 | 250220140 | 23673 |
| CHL1_PRMT5i_72hours_replicate1 | 52590095 | 355772547 | 22992 |
| CHL1_PRMT5i_72hours_replicate2 | 51944543 | 352381305 | 23296 |
| CHL1_CDK4/6i_6days_replicate1 | 44562890 | 204237467 | 23707 |
| CHL1_CDK4/6i_6days_replicate2 | 38032201 | 235212234 | 23848 |
