# Supplementary material for: PRMT5 and CDK4/6 inhibition result in distinctive patterns of alternative splicing in melanoma
Source: PLoS One. 2023 Nov 2;18(11):e0292278. doi: 10.1371/journal.pone.0292278 (PMC10621831; doi:10.1371/journal.pone.0292278)
Supplement: S2 Table — (DOCX) [file pone.0292278.s002.docx]

Table S2. Number of differentially expressed genes in CHL1 and A375 cells under different treatments.

|  | **CHL1** | | | **A375** | | |
| --- | --- | --- | --- | --- | --- | --- |
|  | **CDK4/6i  (72hr)** | **CDK4/6i  (6 days)** | **PRMT5i  (72hr)** | **CDK4/6i  (72hr)** | **CDK4/6i  (6 days)** | **PRMT5i  (72hr)** |
| Up-regulated | 445 | 1689 | 5134 | 1710 | 4876 | 252 |
| Down-regulated | 498 | 1611 | 5754 | 1409 | 4601 | 423 |
| Total | 943 | 3300 | 10888 | 3119 | 9477 | 675 |
