# Supplementary material for: PRMT5 and CDK4/6 inhibition result in distinctive patterns of alternative splicing in melanoma
Source: PLoS One. 2023 Nov 2;18(11):e0292278. doi: 10.1371/journal.pone.0292278 (PMC10621831; doi:10.1371/journal.pone.0292278)
Supplement: S3 Table — (DOCX) [file pone.0292278.s003.docx]

S3 Table. Numbers of differential splicing events of each type detected by rMATs at each time point post-treatment for CHL1 and A375 cells.

| **Cell line** | | **Sample (vs DMSO control)** | **A3SS** | **A5SS** | **MXE** | **RI** | **SE** | **Total** |
| --- | --- | --- | --- | --- | --- | --- | --- | --- |
| CHL1 | CDK4/6i 72hours | | 158 | 166 | 339 | 202 | 876 | 1741 |
| CHL1 | PRMT5i 72hours | | 676 | 929 | 2453 | 2102 | 4683 | 10843 |
| CHL1 | CDK4/6i 6days | | 197 | 194 | 413 | 299 | 1288 | 2391 |
| A375 | CDK4/6i 72hours | | 264 | 270 | 356 | 413 | 1318 | 2621 |
| A375 | PRMT5i 72hours | | 457 | 641 | 585 | 1013 | 3172 | 5868 |
| A375 | CDK4/6i 6days | | 412 | 417 | 1242 | 828 | 2005 | 4904 |
