# Supplementary material for: PRMT5 and CDK4/6 inhibition result in distinctive patterns of alternative splicing in melanoma
Source: PLoS One. 2023 Nov 2;18(11):e0292278. doi: 10.1371/journal.pone.0292278 (PMC10621831; doi:10.1371/journal.pone.0292278)
Supplement: S4 Table — (DOCX) [file pone.0292278.s004.docx]

Table S4. MDM4 skipped exon events of exon number 6 detected by rMATS.

| Cell | Treatment | time | exonStart_0base | exonEnd | IncFormLen | SkipFormLen | PValue | FDR | IncLevel1 | IncLevel2 | IncLevelDifference |
| --- | --- | --- | --- | --- | --- | --- | --- | --- | --- | --- | --- |
| A375 | CDK4/6i | 6 day | 204506586 | 204506625 | 173 | 134 | 0.006499 | 0.157922 | 0.181,0.195 | 0.59,0.311 | -0.263 |
| A375 | CDK4/6i | 6 day | 204506557 | 204506625 | 202 | 134 | 0.038226 | 0.465248 | 0.714,0.465 | 0.838,0.694 | -0.176 |
| A375 | CDK4/6i | 72hr | 204506557 | 204506625 | 202 | 134 | 0.229816 | 1.000000 | 0.719,0.624 | 0.776,0.726 | -0.08 |
| A375 | CDK4/6i | 72hr | 204506586 | 204506625 | 173 | 134 | 0.593456 | 1.000000 | 0.47,0.225 | 0.256,0.326 | 0.056 |
| A375 | PRMT5i | 72hr | 204506557 | 204506625 | 202 | 134 | 0.000000 | 0.000000 | 0.276,0.103 | 0.776,0.726 | -0.561 |
| A375 | PRMT5i | 72hr | 204506586 | 204506625 | 173 | 134 | 0.001044 | 0.032784 | 0.134,0.082 | 0.256,0.326 | -0.183 |
| CHL1 | CDK4/6i | 6 day | 204506586 | 204506625 | 173 | 134 | 0.027396 | 0.428781 | 0.069,0.0 | 0.063,0.114 | -0.054 |
| CHL1 | CDK4/6i | 6 day | 204506557 | 204506625 | 202 | 134 | 0.739397 | 1.000000 | 0.307,0.176 | 0.224,0.296 | -0.019 |
| CHL1 | CDK4/6i | 72hr | 204506586 | 204506625 | 173 | 134 | 0.486474 | 1.000000 | 0.058,0.072 | 0.063,0.114 | -0.023 |
| CHL1 | CDK4/6i | 72hr | 204506557 | 204506625 | 202 | 134 | 0.602313 | 1.000000 | 0.347,0.236 | 0.224,0.296 | 0.031 |
| CHL1 | PRMT5i | 72hr | 204506557 | 204506625 | 202 | 134 | 0.000003 | 0.000272 | 0.04,0.097 | 0.224,0.296 | -0.192 |
| CHL1 | PRMT5i | 72hr | 204506586 | 204506625 | 173 | 134 | 0.017633 | 0.196388 | 0.024,0.024 | 0.063,0.114 | -0.065 |
