# Supplementary figures and images for: PRMT5 and CDK4/6 inhibition result in distinctive patterns of alternative splicing in melanoma
Source: PLoS One. 2023 Nov 2;18(11):e0292278. doi: 10.1371/journal.pone.0292278 (PMC10621831; doi:10.1371/journal.pone.0292278)

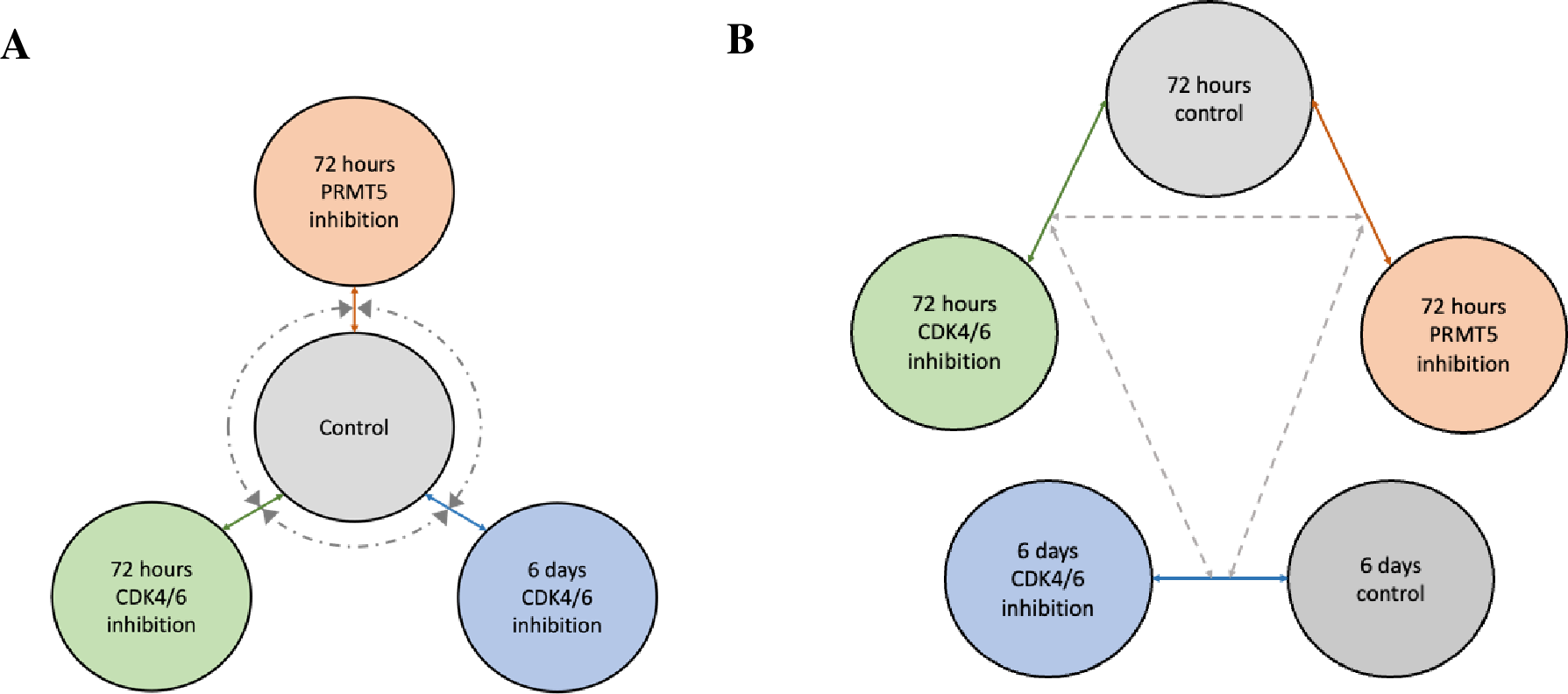

Supplement: S1 Fig — (TIF) [file pone.0292278.s005.tif]

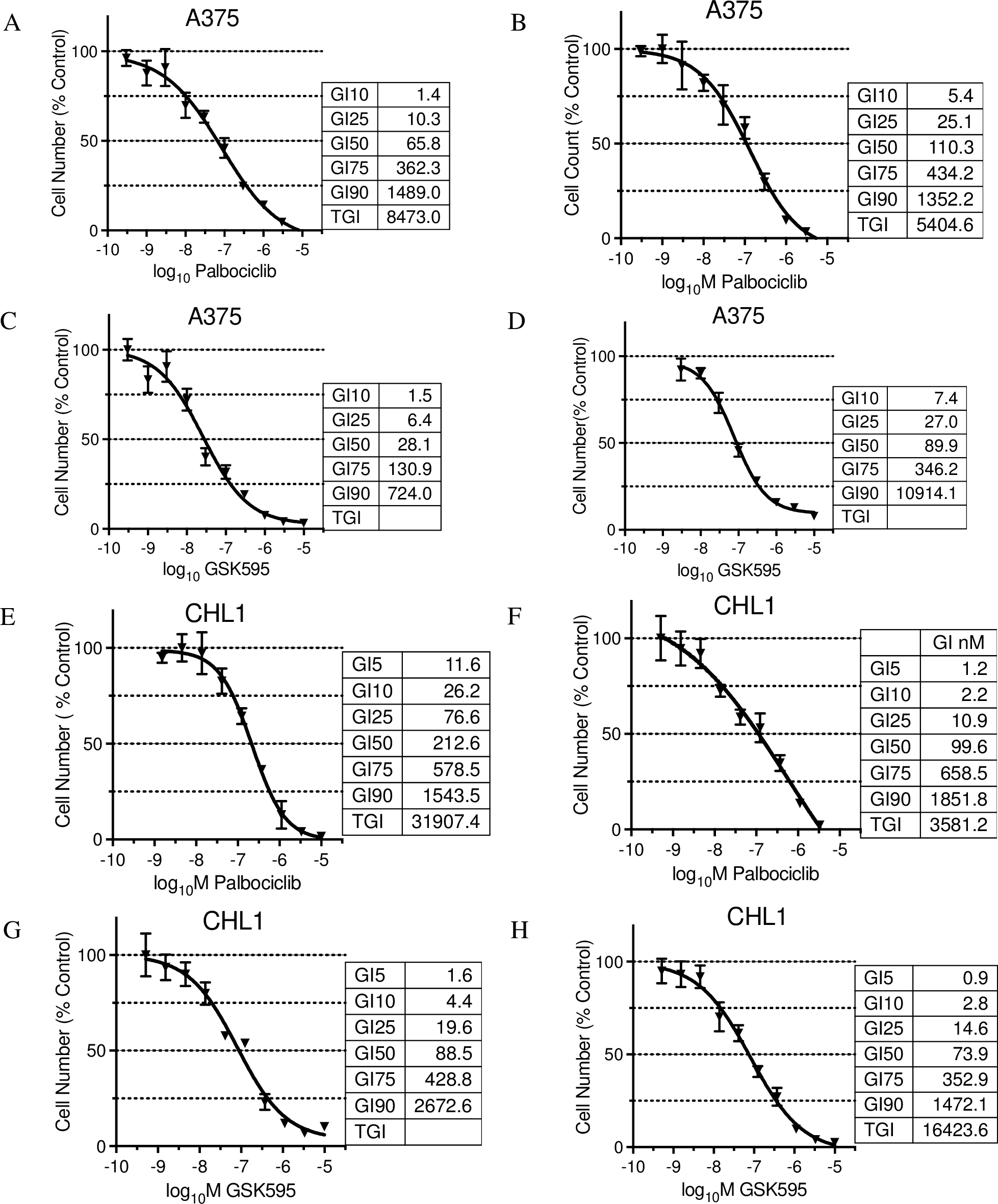

Supplement: S2 Fig — Does response curve of (A,B) Palbociclib treated A375 cell, (C,D) GSK595 treated A375 cell, (E,F) Palbociclib treated CHL1 cell and (G,H) GSK595 treated CHL1 cell. (TIF) [file pone.0292278.s006.tif]

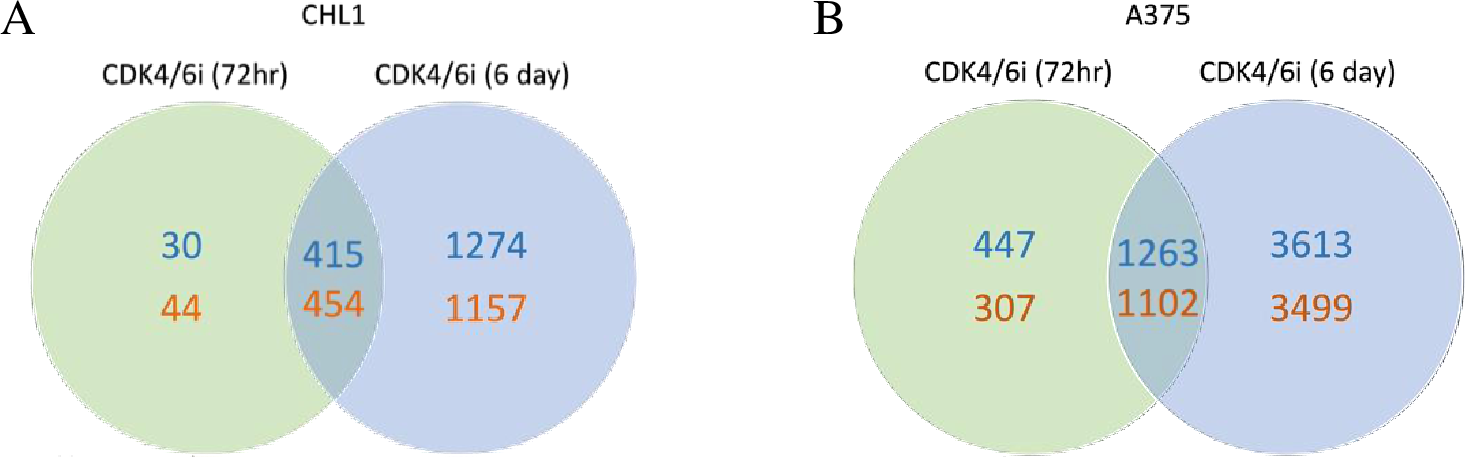

Supplement: S3 Fig — Upregulated (blue) and downregulated (orange) DEGs between CDK4/6i samples in (A) CHL1 and (B) A375. (TIF) [file pone.0292278.s007.tif]

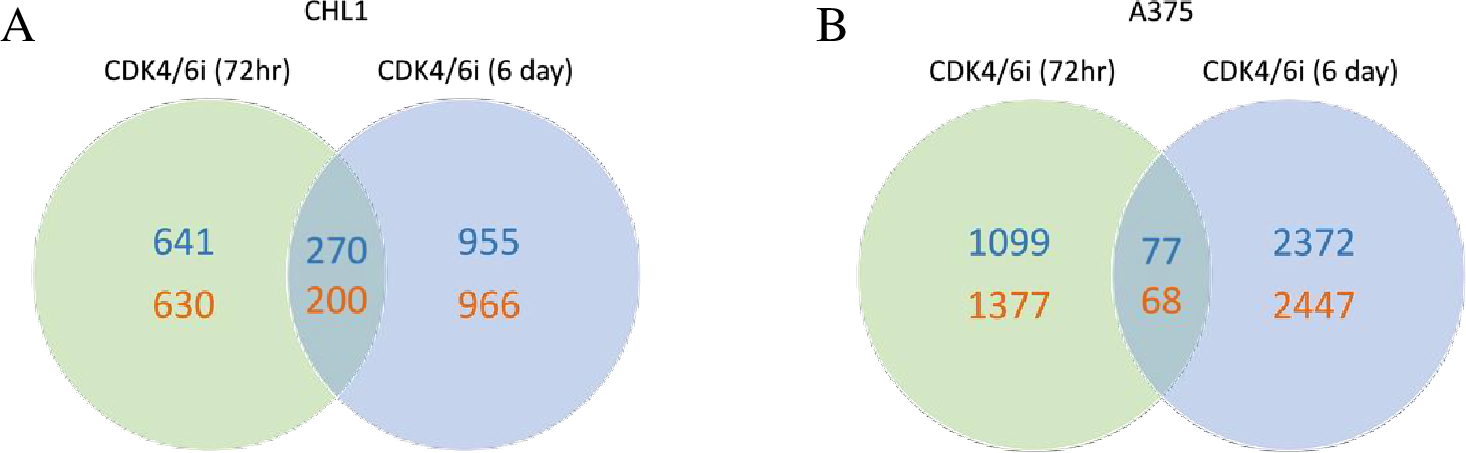

Supplement: S4 Fig — Upregulated (blue) and downregulated (orange) DSEs between CDK4/6i samples in (A) CHL1 and (B) A375. (TIF) [file pone.0292278.s008.tif]

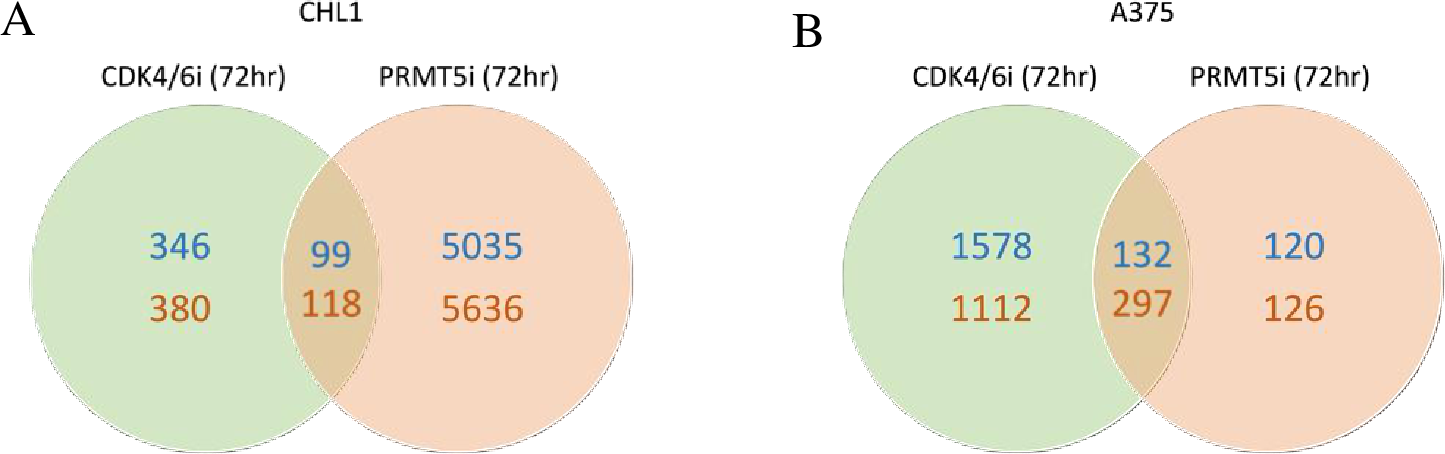

Supplement: S5 Fig — Numbers of up-regulated (blue) and down-regulated (orange) differentially expressed genes at 72hrs of CDK4i and PRMT5 inhibition in (A) CHL1 and (B) A375. (TIF) [file pone.0292278.s009.tif]

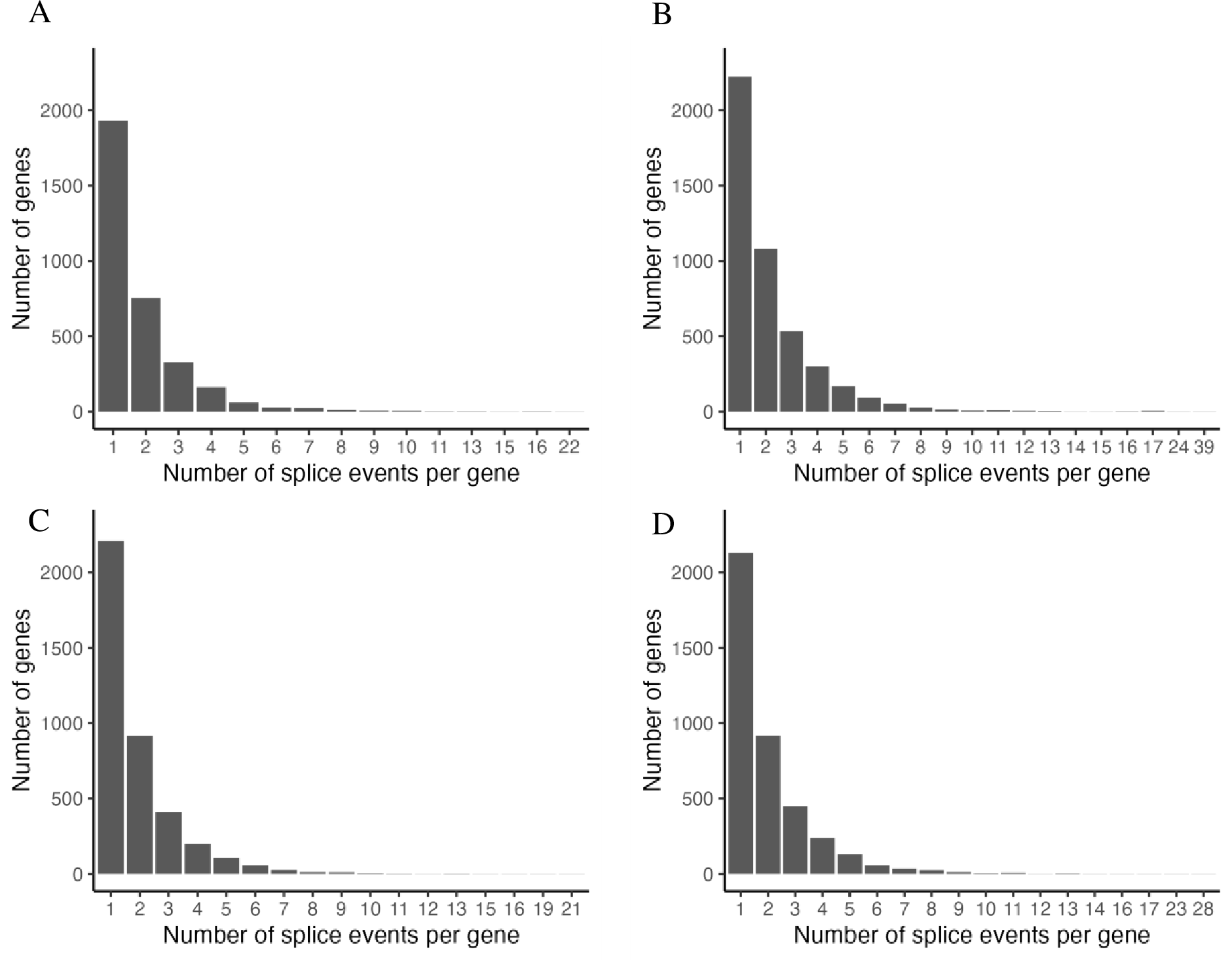

Supplement: S6 Fig — Number of splicing events per gene for (A) 6 days CDK4/6i and (B) 72 hours PRMT5i in CHL1 cells and for (C) 6 days CDK4/6i and (D) 72 hours PRMT5i in A375 cells. (TIF) [file pone.0292278.s010.tif]

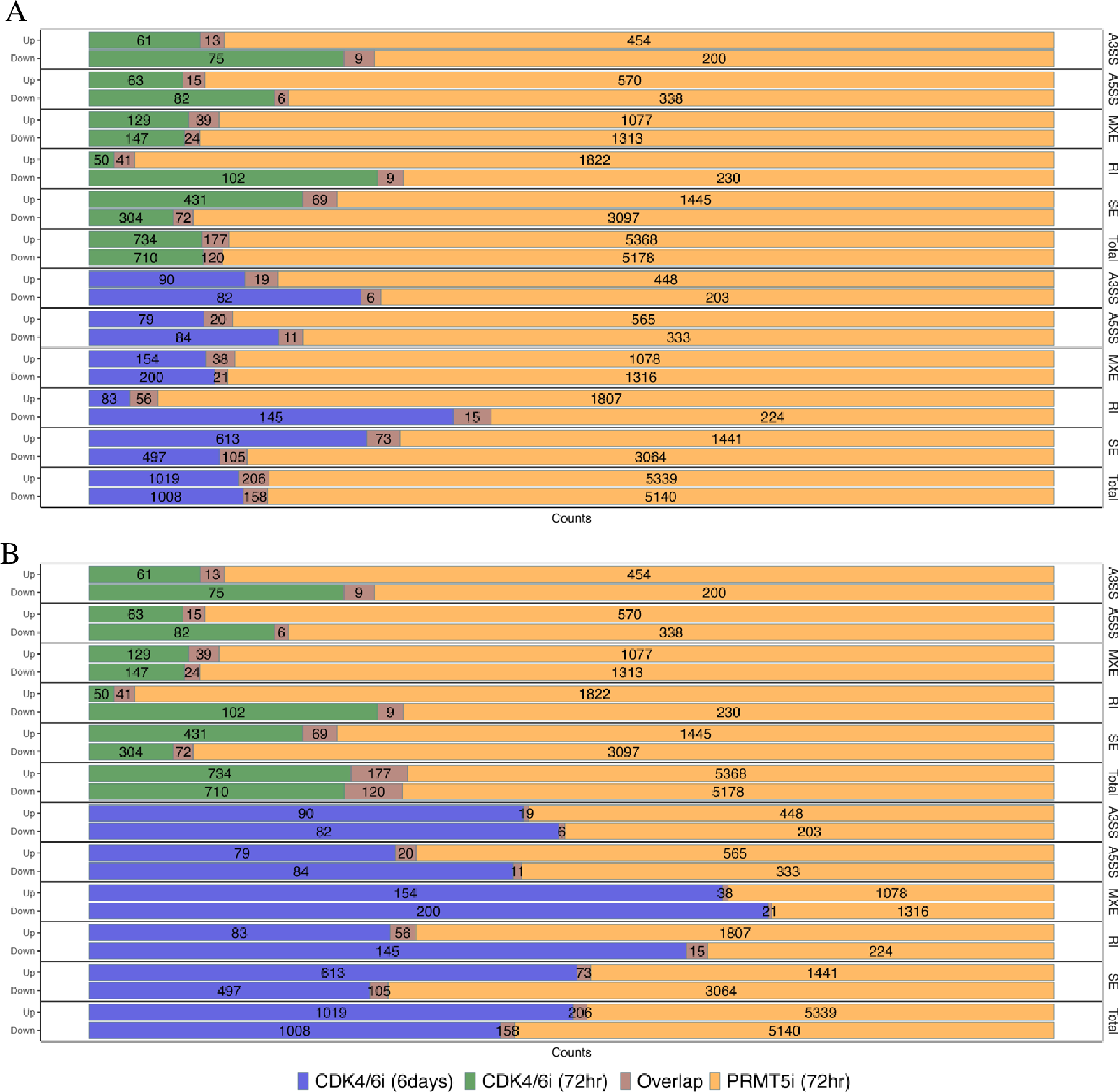

Supplement: S7 Fig — Numbers of up-regulated (blue) and down-regulated (orange) differentially spliced events between 72 hours CDK4/6i, 6 days CDK4/6i and PRMT5 inhibition in (A) CHL1 and (B) A375. (TIF) [file pone.0292278.s011.tif]

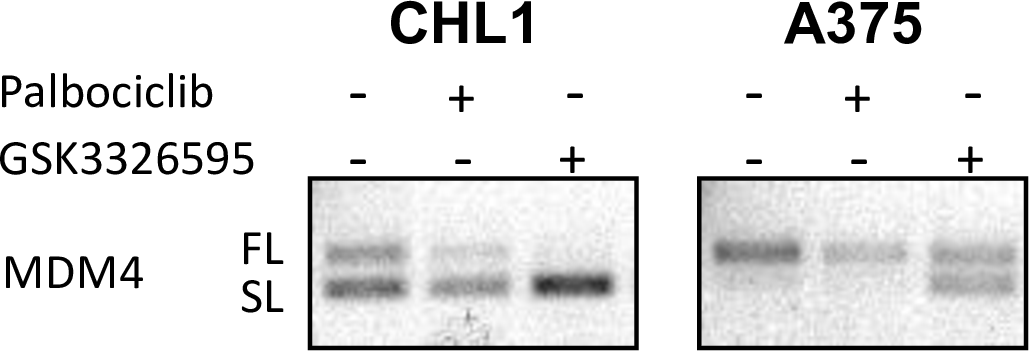

Supplement: S8 Fig — (TIF) [file pone.0292278.s012.tif]

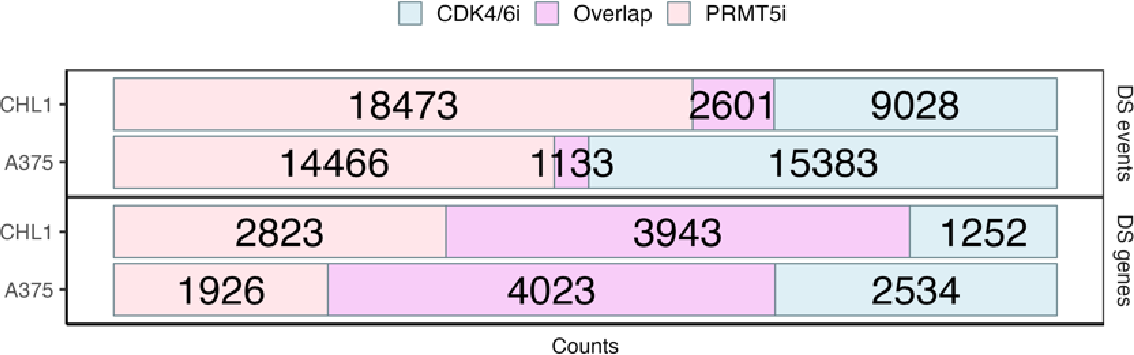

Supplement: S9 Fig — (TIF) [file pone.0292278.s013.tif]

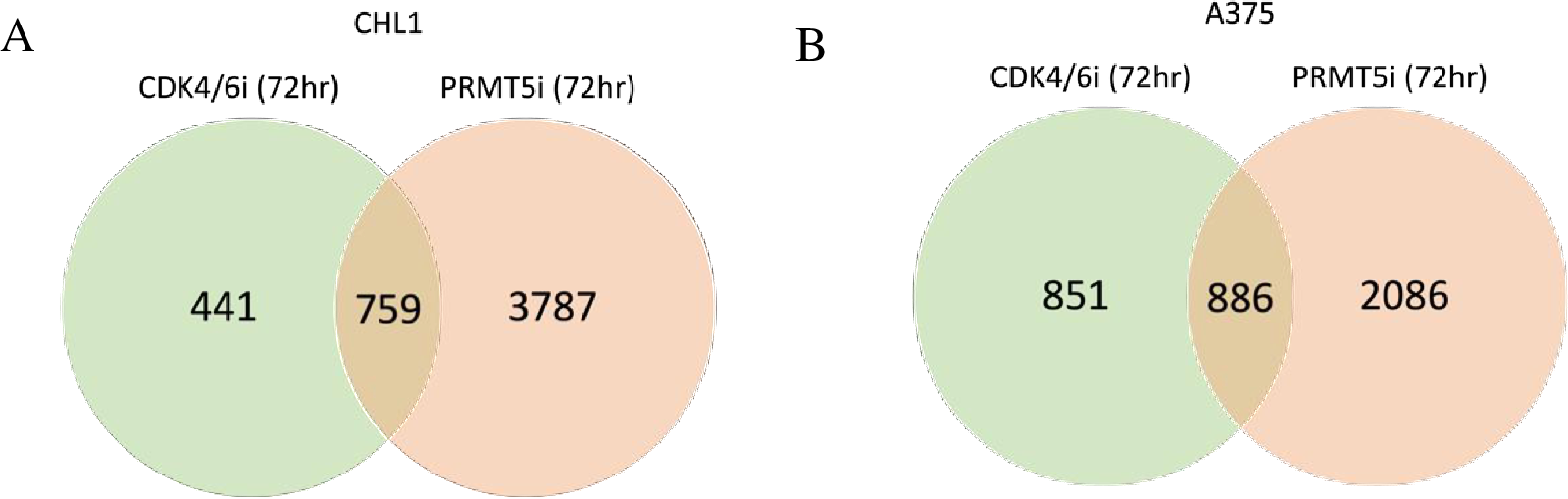

Supplement: S10 Fig — Overlap in differentially spliced genes (DSG) for between72 hours CDK4/6i and PRMT5 inhibition for (A) CHL1 and (B) for A375. (TIF) [file pone.0292278.s014.tif]

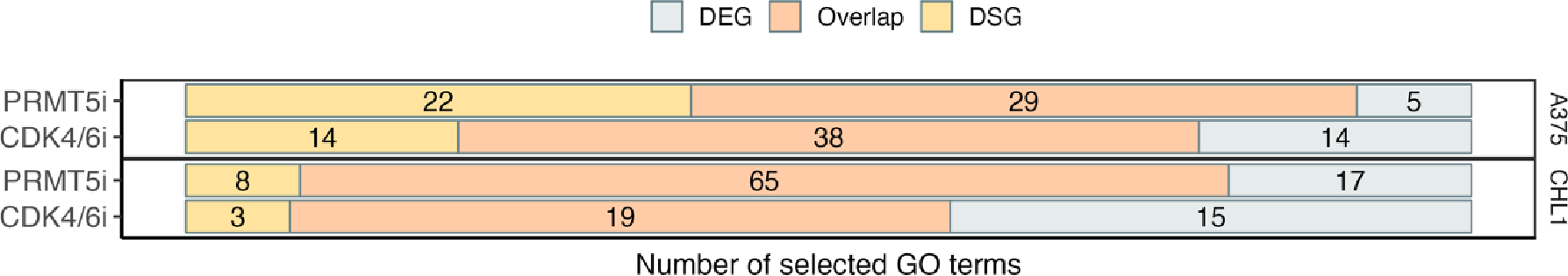

Supplement: S11 Fig — (TIF) [file pone.0292278.s015.tif]

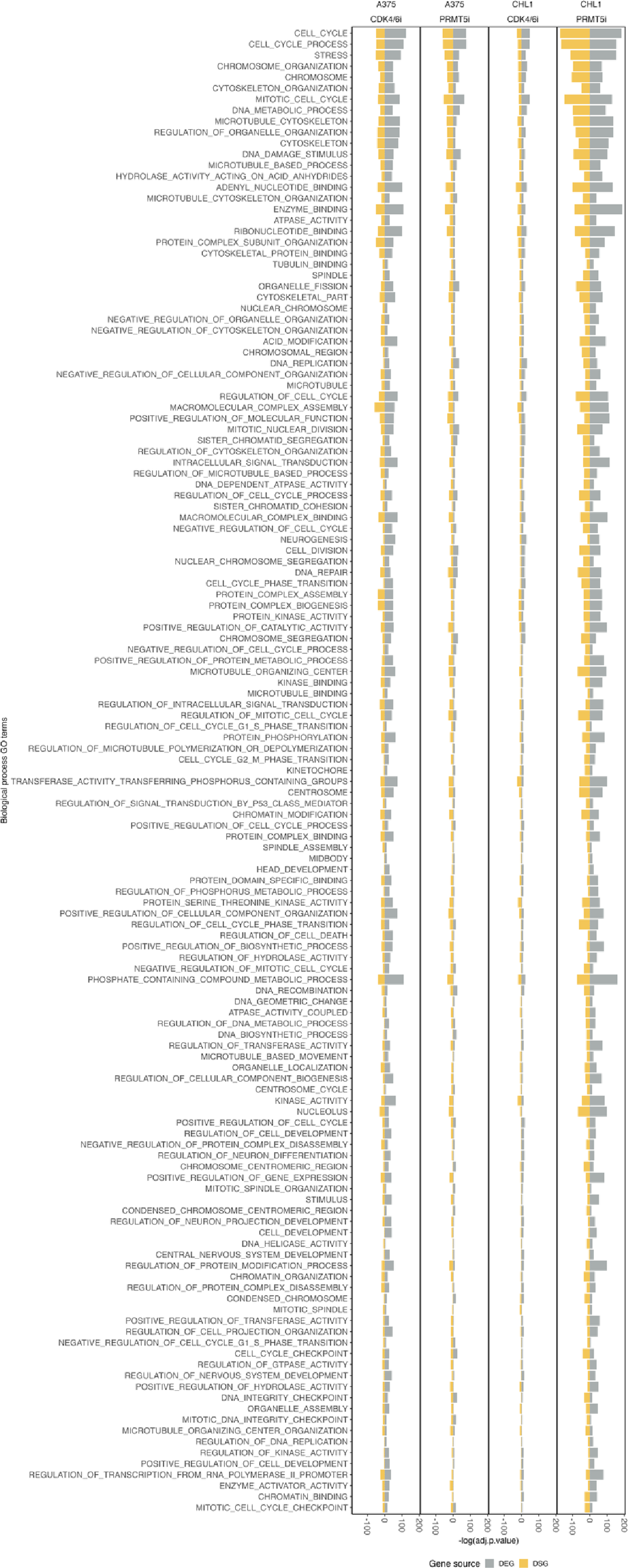

Supplement: S12 Fig — (TIF) [file pone.0292278.s016.tif]
