# Supplementary material for: PRMT5 and CDK4/6 inhibition result in distinctive patterns of alternative splicing in melanoma
Source: PLoS One. 2023 Nov 2;18(11):e0292278. doi: 10.1371/journal.pone.0292278 (PMC10621831; doi:10.1371/journal.pone.0292278)
Supplement: Attachment — Submitted filename: 6_PRMT5_splicing.docx [file pone.0292278.s017.docx]

PRMT5 and CDK4/6 inhibition results in distinctive patterns of alternative splicing in melanoma

In a study by Chan et al., the effects of CDK4/6 inhibitor and PRMT5 on gene expression and alternative splicing were examined in melanoma cells (CHL1 and A375). RNAseq analysis revealed unique gene expression and splicing patterns for each treatment, as well as overlapping and distinct Gene Ontology terms associated with differentially expressed genes and alternatively spliced genes. However, the study lacks experimental evidence linking these results to the biological effects of the treatments in melanoma. The authors suggest a synergistic effect of the combined treatment, but the study does not include a group treated with both inhibitors. To improve the manuscript, the following questions should be addressed:

1. What are the biological phenotypes of CHL1 and A375 cells after treatment with CDK4/6i or PRMT5i, or both? Why was a group treated with both inhibitors not included in the study?
2. Which differentially expressed genes and alternatively spliced genes, as well as their associated Gene Ontology terms, were linked to the biological effects of each treatment or the combined treatment? What experimental evidence supports the expression and splicing of these genes and their association with the biological effects of the treatments?
3. Was MDM4 expression/splicing identified in RNAseq analyses, considering the known interaction between PRMT5 and MDM4 in response to CDK4/6i?
4. What was the rationale for analyzing the results from CHL1 and A375 cells separately, and how many differentially expressed genes and alternatively spliced genes, as well as their associated Gene Ontology terms, overlapped or were distinct between the two cell lines? Which of these genes and GO terms are linked to the biological effects of the treatments?
