# Supplementary material for: PRMT5 and CDK4/6 inhibition result in distinctive patterns of alternative splicing in melanoma
Source: PLoS One. 2023 Nov 2;18(11):e0292278. doi: 10.1371/journal.pone.0292278 (PMC10621831; doi:10.1371/journal.pone.0292278)
Supplement: Attachment — Submitted filename: Chan_etal_responses_to_reviewers.docx [file pone.0292278.s018.docx]

**Response to Reviewer Comments**

For resubmission of the manuscript from Chan et al, titled ‘*PRMT5 and CDK4/6 inhibition result in distinctive patterns of alternative splicing in melanoma*’

We thank our reviewer for the insightful and constructive comments. The manuscript has been revised extensively based on these comments, which includes additional information and reorganization of the results to provide greater clarity. Details of all revisions can be found in ‘ChanEtal_PlosOne_Major_changes_for_resubmission.docx’.

1.	What are the biological phenotypes of CHL1 and A375 cells after treatment with CDK4/6i or PRMT5i, or both? Why was a group treated with both inhibitors not included in the study?

The strongest change in biological phenotype observed under CDK4/6 and PRMT5 inhibition in both lines was reduction in proliferation, as described in the first section of the Results (page 16, lines 225-230). We have now provided proliferation curves to support this observation, in the new Supplementary Figure S2.

Examining the effects of the combination of both inhibitors is beyond the scope of this study. Our main intent is to determine the degree of overlap in alternative splicing events induced by CDK4/6 and PRMT5 inhibition to investigate whether CDK4/6 may control splicing independently of PRMT5, which our results indicate it does. Experiments from the Sheppard laboratory indicate the combination further reduces proliferation to a point where it could be difficult to harvest enough cells for RNA-sequencing at a depth that permits robust splicing analysis, creating a practical hurdle as well.

2.	Which differentially expressed genes and alternatively spliced genes, as well as their associated Gene Ontology terms, were linked to the biological effects of each treatment or the combined treatment? What experimental evidence supports the expression and splicing of these genes and their association with the biological effects of the treatments?

The Gene Ontology term enrichment results presented in Figure 4 show a clear, consistent and strong enrichment for genes involved in the cell cycle for both differentially expressed genes (DEGs) and differentially spliced genes (DSGs). Throughout the manuscript we highlight individual DEGs and DSGs annotated as having well-established links in the literature to the cell cycle, such as CDK12 and E2F family members, as well as MDM2 and MDM4.

3.	Was MDM4 expression/splicing identified in RNAseq analyses, considering the known interaction between PRMT5 and MDM4 in response to CDK4/6i?

Yes, skipping of exon 6 in MDM4 was consistently observed in all samples, albeit only at the more permissive threshold of unadjusted p-value < 0.05 in some samples. These results are now shown in new Supplementary Table S4 and supported by PCR amplification of MDM4 in the region containing exon 6 in CHL1 and A375 after CDK4/6 and PRMT5 inhibition (new Supplementary Figure S8). Even at the more relaxed significance threshold of unadjusted p-value < 0.05, a majority of differential splice events are unique to a single treatment (new Supplementary Figure S9).

4.	What was the rationale for analyzing the results from CHL1 and A375 cells separately, and how many differentially expressed genes and alternatively spliced genes, as well as their associated Gene Ontology terms, overlapped or were distinct between the two cell lines? Which of these genes and GO terms are linked to the biological effects of the treatments?

Given the heterogeneity in melanoma cell lines, inclusion of results from both CHL1 and A375 were intended to provide biological validation and support the notion that our findings are more broadly applicable to melanoma in general. By reorganizing the Results section of the manuscript as we have, we aimed to make the similarities between the two lines more apparent and easily recognizable.

As mentioned above, the primary trend in GO terms enrichment is strong representation of cell cycle and division related terms, consistent with the strong effects of each treatment on cell proliferation.

We hope the revisions outlined here satisfy our reviewer’s concerns and improves overall clarity and comprehension for readers.
